# Biotechnological Production of the Sunscreen Pigment Scytonemin in Cyanobacteria: Progress and Strategy

**DOI:** 10.3390/md19030129

**Published:** 2021-02-27

**Authors:** Xiang Gao, Xin Jing, Xufeng Liu, Peter Lindblad

**Affiliations:** 1 School of Food and Biological Engineering, Shaanxi University of Science & Technology, Xi’an 710021, China; xinjing2020@sust.edu.cn; 2 Microbial Chemistry, Department of Chemistry-Ångstrom, Uppsala University, Box 523, 751 20 Uppsala, Sweden; xufeng.liu@kemi.uu.se

**Keywords:** cyanobacteria, sunscreen pigments, scytonemin, metabolic engineering

## Abstract

Scytonemin is a promising UV-screen and antioxidant small molecule with commercial value in cosmetics and medicine. It is solely biosynthesized in some cyanobacteria. Recently, its biosynthesis mechanism has been elucidated in the model cyanobacterium *Nostoc punctiforme* PCC 73102. The direct precursors for scytonemin biosynthesis are tryptophan and *p*-hydroxyphenylpyruvate, which are generated through the shikimate and aromatic amino acid biosynthesis pathway. More upstream substrates are the central carbon metabolism intermediates phosphoenolpyruvate and erythrose-4-phosphate. Thus, it is a long route to synthesize scytonemin from the fixed atmospheric CO_2_ in cyanobacteria. Metabolic engineering has risen as an important biotechnological means for achieving sustainable high-efficiency and high-yield target metabolites. In this review, we summarized the biochemical properties of this molecule, its biosynthetic gene clusters and transcriptional regulations, the associated carbon flux-driving progresses, and the host selection and biosynthetic strategies, with the aim to expand our understanding on engineering suitable cyanobacteria for cost-effective production of scytonemin in future practices.

## 1. Introduction

Cyanobacteria are photoautotrophic prokaryotes that can directly convert CO_2_ into organic compounds using solar energy. Cyanobacteria possess relatively small genomes and can grow with minimal nutrient requirement. Engineering cyanobacteria offers an attractive approach to drive carbon flux to the biosynthesis of fuels, chemicals, medicines, plant secondary metabolites, and other value-added products [1,2,3,4,5].

Cyanobacteria naturally grow in diverse habitats including fresh water, oceans, alkaline waters, cold deserts, polar regions and hot springs. To cope with the extreme and changeable environments, cyanobacteria have developed a wide range of adaptive mechanisms. Some cyanobacteria are directly exposed to strong sunlight in native habitats, such as those dwelling in intertidal mats or on desert soil surfaces [6,7,8]. Intense ultraviolet (UV) radiation can cause cell damage by both direct effects on proteins and nucleic acids and indirect effects via the induced reactive oxygen species [9]. To eliminate the deleterious effects of UV radiation, one of the most important strategies in some cyanobacteria is to generate UV-absorbing compounds, such as mycosporine-like amino acids (MAAs) and scytonemin [10]. Both compounds are located in the exopolysaccharide matrix and constitute the first line of defense against UV penetration and subsequent damage [11,12,13]. Multiple estimates from the fossil record of cyanobacteria and relaxed molecular clock models suggest a minimum age for the evolutionary advent of scytonemin at around 2.1 billion years [14].

Scytonemin is naturally biosynthesized in some cyanobacteria and has a great prospect in cosmetic and biomedical application. However, as far as we know, enhanced production of this compound in cyanobacteria via metabolic engineering has not been reported. To exploit the maximal potential of engineering cyanobacterial hosts for scytonemin biosynthesis is the next important step. In this review, we summarized the research progresses regarding scytonemin, placing emphasis on its biosynthetic route and the metabolic engineering technologies from cyanobacteria and other microorganisms, with the aim to expand our understanding on metabolically engineering of cyanobacterial hosts for cost-effective production of scytonemin in future practices.

## 2. Functional Roles of Scytonemin 

### 2.1. Molecular Structure and UV Absorption 

**Figure 1:**
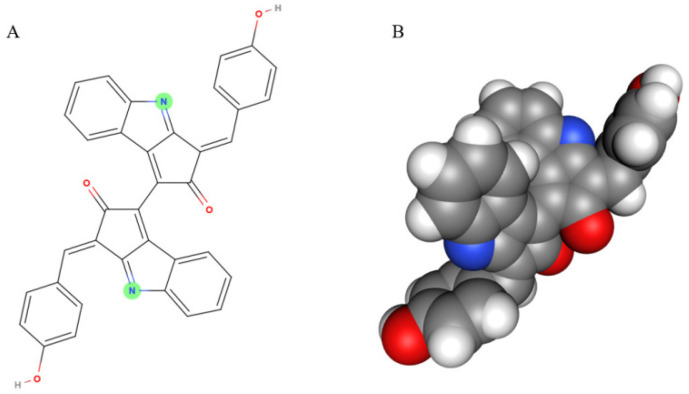
The scytonemin molecule (**A**) and its 3D model (**B**). Two images are generated by the MolView program (https://molview.org/, accessed on 20 August 2020).

Scytonemin is a lipid soluble and yellow-brown pigment exclusively found in some cyanobacteria [11]. This compound is a dimer composed of indolic and phenolic subunits symmetrically connected through a carbon–carbon bond (Figure 1). Scytonemin has oxidized (yellow; Mw 544 Da) and reduced (red; Mw 546 Da) forms and also three derivatives, dimethoxyscytonemin, tetramethoxyscytonemin, and scytonin [15,16].

The solar UV radiation can be divided into UV-A (315–400 nm), UV-B (280–315 nm), and UV-C (100–280 nm). UV-C does not reach the earth’s surface owing to stratospheric ozone. The remaining UV that reaches the surface of the earth consists of 95% UV-A and 5% UV-B [17]. Scytonemin can absorb the three UV radiations, but mainly the UV-A, and can prevent about 90% of UV-A from entering the cells [11,18,19]. It can also provide substantial protection against UV-B and UV-C damage [20,21]. The absorption in the UV-C range implies that scytonemin is an ancient pigment possibly evolved during the Precambrian [20]. Scytonemin has an in vivo maximum absorption at 370 nm and an in vitro maximum absorption at 384 nm [11,22].

### 2.2. Solubility and Stability

Scytonemin does not dissolve in water, but dissolves readily in lipids. In liquid suspension culture of *Nostoc flagelliforme*, yellow-brown scytonemin was observed to be tightly associated with the released exopolysaccharides [23]. Scytonemin was usually extracted from samples with solvents such as 100% acetone or 100% ethyl acetate [11], 100% acetonitrile [24], 80% tetrahydrofuran [13], and methanol:ethyl acetate (1:1, *v/v*) [9,21]. In addition, we found scytonemin was soluble in *N,N*-dimethylformamide and the polyvinylpyrrolidone K30 aqueous solution. Its solubility in polyvinylpyrrolidone K30 solution (e.g., 1% aqueous solution) may facilitate its reaction with other water-soluble compounds.

It was reported that scytonemin showed high stability against the abiotic stress conditions of UV-B (0.78 W·m^−2^), heat (60 °C), and strong oxidizing agent (0.25% H_2_O_2_) for 1 h [25]. After continuous exposure to 55 W·m^−2^ visible light or 5 W·m^−2^ UV-A radiation for 50 days, the original scytonemin content in dry *Nostoc punctiforme* samples remained at 93% and 84%, respectively, while chlorophyll α content decreased to an undetectable level by day 10 under both conditions [26]. Its stability was also evidenced by its preservation in the lake sedimentary record [27]. The abundant preservation of scytonemin in deep sea sediments indicates that scytonemin is resistant to degradation during erosion and transport [28]. For this reason, scytonemin is regarded as one of the key biomarkers in both paleoclimatological reconstructions and in terrestrial extreme environments [29,30,31]. 

### 2.3. Cellular Distribution and Content

**Figure 2:**
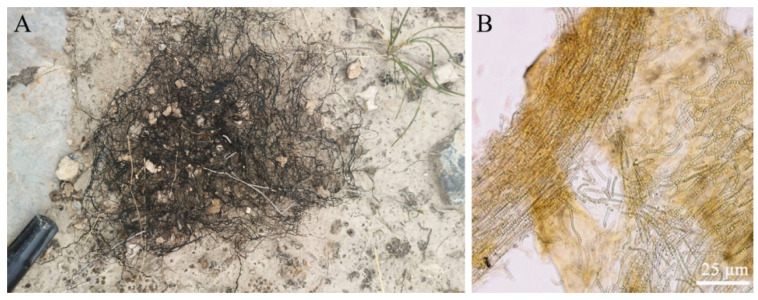
The filamentous colonies (filaments) of *Nostoc flagelliforme* in native habitats (**A**) and the microscopic observation of a crushed filament (**B**). Yellow-brown scytonemin is distributed throughout a filament as shown in (**B**).

Scytonemin is distributed in the exopolysaccharide sheath of more than 300 cyanobacteria, including *Nostoc*, *Scytonema*, *Calothrix*, *Lyngbya*, *Rivularia*, and *Chlorogloeopsis* [30,32,33]. It is predominantly located in the surface layer of samples. Light microscopy sections of *N. flagelliforme* clearly showed the distribution of yellow-brown scytonemin in the peripheral region of exopolysaccharide sheath [13]. Figure 2 shows the distribution of scytonemin in *N. flagelliforme* filaments. CARS microscopy of scytonemin in laboratory-cultivated *Nostoc commune* further imaged its distribution in the surface layer of the sheath after UV-A radiation [34]. In terrestrial cyanobacterial mats or crusts, cyanobacterial upper layer usually shows high amounts of scytonemin, conferring protection to the cells beneath against UV damage [7,30,35,36].

Scytonemin could constitute up to 5% of cyanobacterial dry weight in the cultured organisms [37]. In some examined cyanobacteria, the contents of scytonemin ranged from 0.08 to 7.98% of dry weight [6]. In *N. commune*, dry weight percentages were estimated to be in an approximate ratio of 10:1:0.1 for exopolysaccharide, MAAs, and scytonemin, respectively [38]. 

### 2.4. Biochemical, Medical and Ecological Values 

Scytonemin has received increasing attention for its biochemical and ecological roles owing to UV-absorption and antioxidant functions, and also for its potential application in cosmetic and pharmaceutical industry as an active molecule. It showed strong antioxidant activity and slow radical scavenging activity in the DPPH (2,2-diphenyl-1-picrylhydrazyl) or ABTS (2,2′-azinobis(3-ethylbenzothiazoline-6-sulfonic acid)) assay [24,25,39]. The dose dependent antioxidant activity of scytonemin was 22% and 52% at concentrations of 0.4 and 0.8 mM, respectively, as compared to that of 0.5 mM ascorbic acid used as a positive control [21]. As a natural sunscreen compound, its application for skin protection has attracted great interest among dermatologists and in cosmetics [40,41].

Medically, scytonemin possesses anti-inflammatory and anti-proliferative properties. It inhibits skin inflammation by blocking the expression of inflammatory mediators, partially through down-regulation of NF-κB activity [42]. Moreover, scytonemin inhibits proliferation of human fibroblasts and endothelial cells by selectively inhibiting kinases [43]. The human polo-like kinase 1 that scytonemin inhibits serves as an attractive target of anti-cancerous drugs [44,45]. Scytonemin can also inhibit the activity of other kinases such as Myt1, cyclin-dependent kinase 1 (cyclin B), checkpoint kinase 1, and protein kinase C [46]. In addition, reduced scytonemin can suppress the human T-lymphoid Jurkat cell growth [47], and the LPS/IFNc-stimulated NO production in murine macrophage RAW264 cells [48]. Therefore, scytonemin is a promising small-molecule drug.

Scytonemin possesses important ecological functions and thus shows the great potential in environmental management. It is often found in the upper layers of microbial mats that thrive in areas exposed to intense sunlight. Its photoprotective function is involved in the following mechanisms: UV-absorption and antioxidant functions; reducing the formation of reactive oxygen species and thymine dimers; alleviating the photosynthetic inhibition; heat dissipation from absorbed UV radiation to increase soil surface temperature [22,25,49,50]. In addition, scytonemin has been proposed to confer cyanobacteria with high tolerance to desiccation [26]. One of the mechanisms may be its potential role in stabilizing the exopolysaccharide matrix [8]. Scytonemin may also interact with other extracellular components in the matrix, such as WspA protein, to indirectly function in the desiccation resistance [38]. Scytonemin and iron can form the iron-complexes that possibly facilitate the survival of cyanobacterial colonies on sandstone rocks [51,52].

## 3. Abiotic Factors Involved in the Induced Biosynthesis of Scytonemin

Although scytonemin is preferentially induced in cyanobacteria by UV radiation, other environmental and nutrient factors have also been reported to incur its induction. In contrast to the strong induction by UV-A radiation, blue, green, or red light has no significant effect on the induction [11]. In *Lyngbya aestuarii*, the content of scytonemin was lowest at zero salinity level and increased with increasing salinity in the culture medium [53]. In conjunction with UV-A radiation, both high temperature and photooxidative conditions caused an increase of scytonemin production in *Chroococcidiopsis*, while increased salt concentration inhibited its scytonemin synthesis [54]. The comparison of scytonemin biosynthesis in three desiccation-tolerant cyanobacterial strains, *N. punctiforme* PCC 73102, *Chroococcidiopsis* CCMEE 5056, and *Chroococcidiopsis* CCMEE 246, showed that in presence of UV-A radiation, the former two produced more scytonemin when experiencing periodic desiccation than when continuously hydrated [26]. The production of scytonemin in *N. punctiforme* PCC 73102 increased 3–7 times in a diazotrophic culture as compared to the non-diazotrophic culture [55]. In nitrogen-deficiency medium, the *N. flagelliforme* culture treated with 0.2 W·m^−2^ UV-B for 30 days produced nearly 5 times more scytonemin than the non-treated samples. It was also observed that a light of 60–90 μmol photons·m^−2^·s^−1^ induced the production of scytonemin in the exopolysaccharides of *N. flagelliforme* culture [23].

The exoploysaccharide plays a major role in protecting cells from environmental stresses, and its biosynthesis is similarly induced by salt stress, high temperature, high light, and UV-B radiation [56]. Considering the localization of scytonemin in the polysaccharide-rich extracellular matrix, it is an interesting question whether the biosynthesis of both compounds is simultaneously regulated or tightly coupled. In contrast to scytonemin, the exopolysaccharide is more widely biosynthesized in prokaryotic microorganisms with seemingly more complex mechanisms [56,57]. Without UV induction, scytonemin was rarely biosynthesized in the *N. flagelliforme* culture [58], but the exopolysaccharide can still be massively biosynthesized [23]. Using a non-scytonemin-producing *N. punctiforme* mutant, it was found that the exopolysaccharide production was more closely related to oxidative stress than UV-A radiation [59]. Thus, ascertaining the UV-specific cis-regulatory element should be crucial for developing preferential biosynthesis of scytonemin in engineered cyanobacteria. 

## 4. Genes for Scytonemin Biosynthesis and Secretion

### 4.1. Gene Clusters for the Direct Biosynthesis

**Figure 3:**
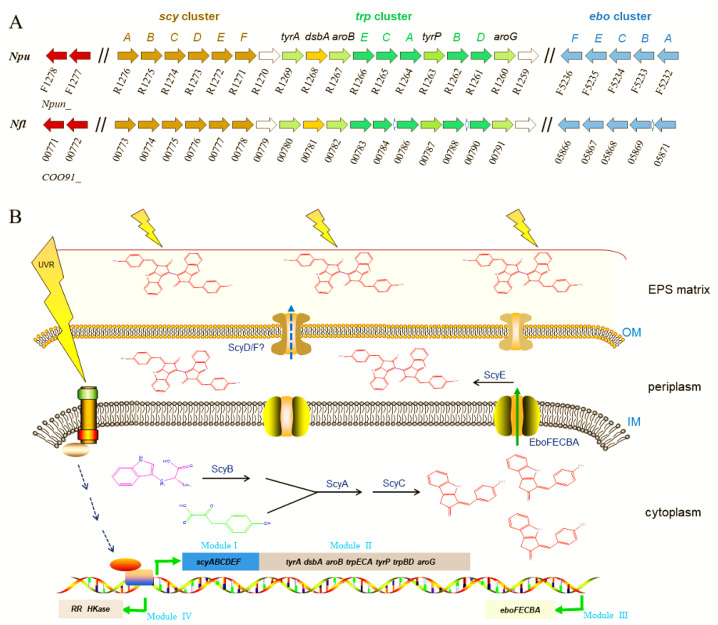
Homologous comparison of scytonemin biosynthesis-related genes between two genetically close *Nostoc* species (**A**) and schematic illustration of UV-induced scytonemin biosynthesis processes in cyanobacteria (**B**). *Npu*, *Nostoc punctiforme*; *Nfl*, *Nostoc flagelliforme*. The gene clusters are distinguished as four modules. Module I, *scyABCDE*F, responsible for scytonemin monomer and dimer biosynthesis. Module II, *tyrA~aroG*, responsible for biosynthesis of aromatic amino acid substrates. Module III, *eboFECBA*, responsible for the export of scytonemin monomer. Module IV, responsible for signal transduction in response to UV radiation. Abbreviations: IM, inner membrane; OM, outer membrane; EPS matrix, exopolysaccharide matrix.

Elucidating the genes for scytonemin biosynthesis is essential for the development of metabolically engineered strains for commercial production of scytonemin. The scytonemin biosynthetic gene cluster (*scy* cluster) was first identified through the analysis of a transposon mutagenesis-generated non-scytonemin-producing mutant of *N. punctiforme* ATCC 29133 (PCC 73102) [60]. This mutation was located within a cluster of 18 open reading frames (*Npun_R1276–R1259*) that were all transcribed in the same direction. Currently, the entire pathway of scytonemin biosynthesis has been basically elucidated, as shown in Figure 3. The genes involved in the biosynthesis, secretion, and regulation processes of scytonemin can be distinguished as four functional sets (or four modules) [61,62,63,64]. Among them, the above-mentioned 18 genes for scytonemin biosynthesis can be divided into two modules: Module I (*scyABCDEF*, *Npun_R1276–R1271*) and module II (*Npun_R1270–R1259*). In module I, ScyABC proteins are involved in catalyzing the formation of the scytonemin monomer within the cells [61], while ScyDEF are thought to be responsible for oxidation and dimerization of the monomer to form the scytonemin dimer in the periplasm [63]. ScyB (Npun_R1275), a leucine dehydrogenase homolog, catalyzes the oxidative deamination of tryptophan (Trp) substrate to yield indole-3-pyruvic acid. ScyA (Npun_R1276), a thiamin diphosphate-dependent enzyme, then mediates the acyloin coupling of indole-3-pyruvic acid and *p*-hydroxyphenylpyruvate (*p*-Hpp), to afford a labile β-ketoacid compound. Subsequently, ScyC (Npun_R1274) catalyzes the cyclization and decarboxylation of the β-ketoacid compound to form a ketone, which is one (auto)oxidation state away from what is called the scytonemin monomer [61,65]. ScyE (Npun_R1272) is essential for catalyzing the final oxidative dimerization of the scytonemin monomer to scytonemin in the periplasm, while ScyD (Npun_R1273) and ScyF (Npun_R1271) seem to not be essential for this catalytic process [63]. Since scytonemin is located in the cyanobacterial exopolysaccharide matrix, particularly its peripheral region [13], an unanswered question is how scytonemin is transported or diffused from the periplasm to the exopolysaccharide matrix for final location. Whether ScyD and/or ScyF play a role in this process remains an open topic.

Scytonemin synthesis requires the aromatic amino acids as building blocks. The genes in module II are predicted to be involved in the biosynthesis of aromatic amino acid substrates [60,66,67]. Except *Npun_R1268* (encoding a DSBA oxidoreductase) and *Npun_R1269* (encoding a prephenate dehydrogenase), other genes in Module II have at least two homologous genes. The redundancy of the aromatic amino acid biosynthetic genes should be beneficial for providing more available substrates for scytonemin biosynthesis.

The *ebo*ABCEF gene cluster (*Npun_F5232–5236*), module III, is responsible for translocation of the scytonemin monomer to the periplasm [64]. They function together, and the absence of any one of them prevents this translocation, leading to cytoplasmic accumulation of the scytonemin monomer [64]. The *ebo* cluster is widespread and conserved in some cyanobacteria, Ceustigmatophytes, Bacteroidetes, and *Leptospira* [68]. An engineered *Escherichia coli* strain that harbored the scytonemin biosynthetic genes could only produce the scytonemin monomer, not the final scytonemin dimer [69]. We speculate that the lack of EboABCEF complex might be a potential reason for this defect in *E. coli*. The *ebo* cluster was also found in *N. flagelliforme* (*COO91_05871–05866*) and non-scytonemin-producing *Nostoc* PCC 7120 (*all0421–0415*). In *Nostoc* PCC 7120, the incompleteness of scytonemin biosynthetic genes, lacking the homologous genes of *Npun_R1268* and *Npun_R1263*, might be one of the key reasons for the incapability in scytonemin biosynthesis in this particular strain.

Two genes that encode a histidine kinase (*Npun_F1277*) and a response regulator (*Npun_F1278*), module IV, serve as a two-component regulatory system responsible for multiple biological processes including the biosynthesis of scytonemin [70]. The deletion of *Npun_F1278* led to the incapability in scytonemin biosynthesis under either UV-A radiation or white light [70]. The transcription of *scyA,* the first gene in *scy* cluster, was measured to be strongly inhibited in the *Npun_F1278* mutant strain. Both Npun_F1277 and Npun_F1278 have multiple paralogs in *N. punctiforme* (e.g., Npun_R3198 and Npun_F4909). Biochemical identification of the specific interaction between the response regulator Npun_F1278 and the transcriptional regulation elements of *scy* cluster as well as other environmental factors-associated effects need to be further elucidated. 

### 4.2. Transcriptional Regulation under UV-A/B Radiation

Various abiotic stresses are involved in the induction of scytonemin biosynthesis. UV-A radiation is the most effective inducer. Understanding the transcription of scytonemin production-associated genes in response to UV-A is important for strategy design in metabolic engineering. Under 0.64 mW·cm^−2^ UV-A radiation for 48 h, transcriptional levels of all the genes in module I (*Npun_R1276–R1271*) and module II (*Npun_R1270–R1259*) increased 44~82% in *N. punctiforme* [67]. During the treatment of 0.5 W·cm^2^ UV-A for 7 days, a dynamic transcription was observed for these genes, mostly reaching a maximum at 48 h, with transcriptional levels elevating 2.8 to 5.2 folds over the control [66]. AroG and AroB (respectively encoded by *Npun_R1260* and *Npun_R1267*) are two major points of regulation in the shikimate pathway [71]. These clues imply that all the six genes of module I for direct scytonemin biosynthesis and the two genes of module II involved in the biosynthesis of aromatic amino acids are critical targets for transcriptional modulation in metabolic engineering. In addition, all the five genes (*Npun_F5232–F5236*) of module III showed a generally subdued transcriptional induction upon the UV-A radiation, with increases ranging from only 1.9 to 3 folds [66]. Both genes (*Npun_F1277* and *Npun_F1278*) of module IV showed reduced or transient transcriptional induction by UV-A radiation [66,67], emphasizing their regulative or signaling role in the biosynthesis of scytonemin.

**Figure 4:**
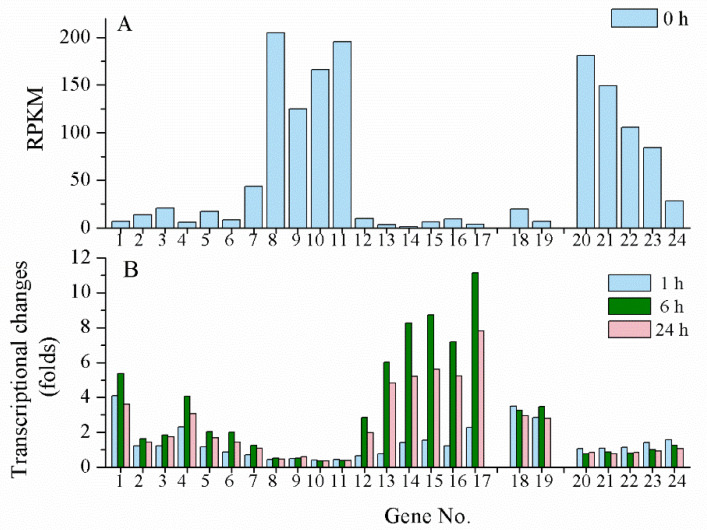
The transcriptional changes of scytonemin biosynthetic and export-associated genes in response to UV-B radiation. The physiologically recovered *Nostoc flagelliforme* samples were subjected to 0.5 W·m^−2^ UV-B radiation for 24 h. (**A**) The basal RPKM values of the genes according to our transcriptomic analysis. (**B**) Transcriptional changes of the genes at 1, 6, and 24 h of UV-B radiation. Genes no.1-17, *COO91_00791–00773* (excluding *COO91_00785* and *COO91_00789*); genes no. 18-19, *COO91_00772–00771*; genes no. 20-24, *COO91_05871–05866* (excluding *COO91_05870*).

UV-B is another effective inducer for scytonemin biosynthesis. However, its biosynthesis gene clusters in *N. flagelliforme* exhibited different induction patterns under UV-B radiation according to our transcriptional analysis data (unpublished). When rewetted *N. flagelliforme* samples were treated by 0.5 W·m^2^ UV-B for 24 h, the RPKM values of the gene clusters *COO91_00783–00779* (similar to *Npun_R1266–R1270* in module II) and *COO91_05871–05866* (similar to *Npun_F5232–F5236* of module III) were much higher than those of other genes before the UV-B treatment (0 h) (Figure 4A), implying their high basal expression levels. However, both clusters were not induced or even inhibited by UV-B (Figure 4B). The gene cluster *COO91_00778–00773* (similar to *Npun_R1271–R1276* of module I) was highly induced in transcriptional level by UV-B, and the two-component regulatory genes *COO91_00772* and *COO91_00771* (similar to *Npun_F1277* and *Npun_F1278* of module IV) and other two genes *COO91_00791* and *COO91_00787* (similar to *Npun_R1260* and *Npun_R1263* in module II, respectively) were also significantly induced (Figure 4B). These results indicate a different transcriptional pattern of scytonemin biosynthetic genes in response to UV-B radiation in *N. flagelliforme*. 

### 4.3. Precursors/Substrates Biosynthesis and Key Catalytic Steps

**Figure 5:**
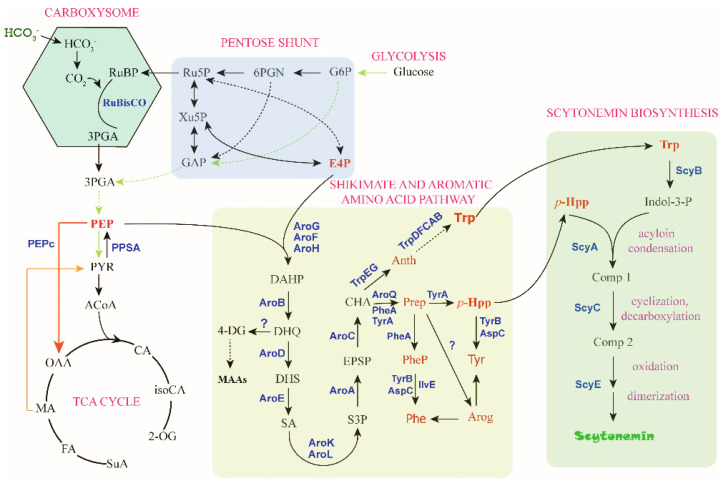
The “C→C” conversion pathway of scytonemin biosynthesis. The direct precursors for scytonemin synthesis are Trp and *p*-Hpp, and the substrates for these two metabolites are PEP and E4P. Metabolite abbreviations: RuBP, ribulose-1,5-bisphosphat; Ru5P, ribulose-5-phosphate; Xu5P, xylulose-5-phosphate; GAP, glyceraldehyde-3-phosphate; 6PGN, 6-phosphate gluconate; G6P, glucose-6-phosphate; E4P, erythrose-4-phosphate. 3PGA, 3-phosphoglycerat; PEP, phosphoenolpyruvate; PYR, pyruvate; ACoA, acetyl-CoA; OAA, oxaloacetate; CA, Citrate; iosCA, iso-citrate; 2-OG, alpha-ketoglutarate; MA, malate; FA, fumarate; SuA, succinyl CoA. DAHP, 3-deoxy-D-arabino-heptulosonate-7-phosphate; DHQ, 3-dehydroquinate; DHS, 3-dehydroshikimate; SA, shikimate; S3P, SA-3-phosphate; EPSP, 5-enolpyruvyl-shikimate 3-phosphate; CHA, chorismate; 4-DG, 4-deoxygadusol; MAAs, mycosporine-like amino acids; Anth, anthranilate; Trp, tryptophan; Prep, prephenate; *p*-HPP, *p*-hydroxyphenylpyruvate; Phe, phenylalanine; Tyr, tyrosine; PheP, phenylpyruvate; Arog, arogenate. Indol-3-P, indole-3 pyruvate; Comp 1, intermediate compound 1; Comp 2, intermediate compound 2. Enzyme abbreviations: RuBisCO, ribulose-1,5-bisphosphate carboxylase/oxygenase; PEPc, PEP carboxylase.

For metabolic engineering of scytonemin production in cyanobacteria, one of the most important steps is to improve the cellular concentrations of the aromatic compound substrates, Trp and *p*-Hpp. Metabolic engineering for production of aromatic amino acids was recently reported in cyanobacteria [72,73], most of related research is yet from other microorganisms, including *E. coli.* To a certain extent, cyanobacterial metabolic engineering can take advantage of the rich toolbox or strategy developed for *E. coli*. Phosphoenolpyruvate (PEP) and erythrose-4-phosphate (E4P) from the central carbon metabolism serve as the substrates for the biosynthesis of Trp and *p*-Hpp, and the biosynthesis process is realized through the shikimate pathway followed by the branched aromatic amino acid pathway with chorismate (CHA) serving as a major branch point intermediate metabolite (Figure 5). This section summarizes the progress regarding the four critical substrates, PEP, E4P, Trp, and *p*-Hpp. 

#### 4.3.1. PEP Biosynthesis 

PEP represents one of the important intermediates in the central carbon metabolism of the cells [74]. In cyanobacteria, CO_2_ is assimilated to generate the building blocks of amino acids and other carbon-containing molecules via the Calvin-Benson-Bassham (CBB) cycle. Ribulose-1,5-bisphosphate carboxylase/oxygenase (Rubisco) catalyzes the addition of CO_2_ to ribulose-1,5-bisphosphate (RuBP), generating two molecules of 3-phosphoglyceric acid (3-PGA) [75]. 3-PGA can diffuse out of the carboxysome and enter into the glycolysis pathway to form PEP. In addition, 3-PGA can also be glycolytically produced from glucose. PEP is then converted to pyruvate by the pyruvate kinase or alternatively converted to oxaloacetate (OAA) by PEP carboxylase (PEPc). PEPc serves as the second major carbon-fixing enzyme in cyanobacteria [76]. Combination of PEPc, malate dehydrogenase and malic enzyme forms a metabolic shunt for the biosynthesis of pyruvate, replenishing the tricarboxylic acid (TCA) cycle [77]. Overexpression of PEPc in *Synechocystis* (*Synechocystis* PCC 6803) resulted in improved succinate production via the reductive TCA cycle and improved 2-oxoglutarate (2-OG) production via the oxidative TCA cycle [78,79]. 

In order to increase the PEP pool, several useful strategies can be employed, including overexpression of PEP-forming enzymes (i.e., PEP synthase and PEP carboxykinase) and inactivation of PEP-degrading enzymes (i.e., pyruvate kinase and PEPc) [78,79,80]. Inhibition of PEPc might be also targeted for maintaining the PEP level, since PEPc can be allosterically inhibited by metabolic effectors, such as malate in several cyanobacteria or after being mutated [81]. A gluconeogenic enzyme phosphoenolpyruvate synthase (PPSA) catalyzes the reverse conversion from pyruvate to PEP [82]. Therefore, enhancement of PPSA should increase the PEP pool. In *E. coli*, the phosphoenolpyruvate-carbohydrate phosphotransferase system (PTS) is responsible for the transport and phosphorylation of sugars, such as glucose, at the expense of PEP [83]. Inactivation of the PTS reduced the PEP consumption [71]. However, this is not necessary for cyanobacterial engineering, since it should be more cost-effective to use the photosynthetically fixed carbon than to use the sugars as carbon source. The excess fixed carbon of cyanobacteria can be stored as glycogen and can be degraded during the dark/light transition [84], which will contribute to buffer the available PEP.

#### 4.3.2. Erythrose-4-Phosphate (E4P) Biosynthesis

E4P is an intermediate in the pentose phosphate pathway (PPP) and the CBB cycle. The PPP is an alternative pathway of glucose metabolism. The function of the PPP includes: Generating NADPH for reductive metabolic reactions within cells, generating ribose-5-phosphate (R5P) as a precursor for nucleotide biosynthesis, and providing E4P as a precursor for aromatic amino acid biosynthesis [85,86]. PPP consists of an oxidative and a non-oxidative phase. In the oxidative phase, NADPH is generated during the oxidative decarboxylation of glucose-6-phosphate (G6P) to form ribulose-5-phosphate (Ru5P). In the non-oxidative phase, Ru5P is converted into R5P or xylulose-5-phosphate (Xu5P). These two five-carbon compounds are then converted to glyceraldehyde 3-phosphate (GAP) and sedoheptulose-7-phosphate (S7P) by transketolase. GAP and S7P are further converted into E4P and fructose 6-phosphate (F6P) by transaldolase. Overexpression of transketolase and/or transaldolase resulted in an increase of the E4P pool [71,87]. E4P can also be directly produced from the catabolism of sedoheptulose-1,7-bisphosphate by the universally conserved glycolytic enzymes ATP-dependent phosphofructokinase and aldolase, when the intracellular level of S7P is high [88].

#### 4.3.3. Trp and p-Hpp Biosynthesis

The aromatic amino acids are not only the building blocks of proteins, but also precursors for a wide range of secondary metabolites in plants and microorganisms. Plants have evolved with interactive plastidial and cytosolic pathways for the biosynthesis of aromatic amino acids [89]. Unlike plants, cyanobacteria exhibit endo-oriented control of the multi-branched aromatic biosynthesis pathway and biosynthesize moderate amounts of aromatic amino acids under natural conditions [90]. 

#### Regulation in the Shikimate Pathway 

The direct substrates for scytonemin biosynthesis, catalyzed by ScyABCDEF in cyanobacteria, are Trp and *p*-Hpp [61]. Both substrates are biosynthesized from PEP and E4P via the shikimate and aromatic amino acid pathway. The shikimate pathway comprises seven enzymatic steps that convert PEP and E4P into CHA [91]. The serial metabolic intermediates are 3-deoxy-d-arabino-heptulosonate-7-phosphate (DAHP), 3-dehydroquinate (DHQ), 3-dehydroshikimate (DHS), shikimate (SA), shikimate-3-phosphate (S3P), 5-enolpyruvylshikimate 3-phosphate (EPSP), and CHA. The most studied enzymes in this pathway are the first and sixth enzyme, respectively, DAHP synthase and EPSP synthase. DAHP synthase is a major point of regulation in the initiation of the shikimate pathway. *E. coli* has three isozymes of DAHP synthase, AroF (catalyzing ~20% of enzymatic activity), AroG (catalyzing ~80% of enzymatic activity), and AroH (catalyzing ~1% of enzymatic activity), each of which is allosterically feedback-regulated by one of the aromatic amino acids, tyrosine (Tyr), phenylalanine (Phe), and Trp [92,93]. EPSP synthase catalyzes the formation of EPSP from PEP and S3P, which is an inhibitory target by the herbicide glyphosate [94]. Other enzymes, DHQ synthase, SA kinase, and CHA synthase, are also rate-limiting enzymes in the shikimate pathway [95,96,97]. 

*E. coli* strains, harboring insensitive mutations of AroG and AroF, showed increased carbon flux toward to SA and aromatic amino acid production through the shikimate pathway [71,87]. In *Synechocystis*, overexpression of the feedback-inhibition-resistant AroG and TyrA enzymes from *E. coli* led to the conversion of an estimated 56% of the total fixed carbon to Phe and Tyr [72]. In addition, the intracellular levels of the three DAHP synthase(s) in *E. coli* are also controlled by transcriptional repression through the repressors TyrR and TrpR, and deletion of *tyrR* and *trpR* alleviated the transcriptional control [98,99,100]. Enhancing the expression of DHQ synthase (encoded by *aroB*) and SA kinase isoenzymes I and II (encoded by *aroK* and *aroL*, respectively) via various genetic engineering approaches resulted in increased aromatic amino acid production [87]. 

Intermediates in the shikimate pathway are also precursors for biosynthesis of diverse secondary metabolites. The shikimate pathway is the predominate route for UV-induced MAA biosynthesis [101]. DHQ is also a possible precursor for biosynthesis of another group of sunscreens, mycosporines and MAAs, via 4-deoxygadusol [102]. In cyanobacteria, MAAs and scytonemin are both effectively induced by UV radiation [13,38], and disruption of the bypass in MAA biosynthesis should be potentially beneficial for scytonemin production. 

#### Regulation in the Aromatic Amino Acid Pathway

CHA is a common precursor for biosynthesis of the three aromatic amino acids, Phe, Tyr, and Trp. In total, five successive enzymatic reactions catalyzed by TrpABCDEFG in *E. coli* lead to the formation of Trp, in which anthranilate synthase (AS) catalyzes the first committed step and is allosterically regulated by Trp [87,103,104]. Transgenic expression of a feedback-resistant AS from *E. coli* improved the Trp production in *Synechocystis* [73]. In *E. coli*, tryptophanase (TnaA) converts Trp to indole, and deletion of *TnaA* gene led to reduced Trp degradation [99,105]. Trp production was also affected by the modification of transport systems. Overexpression of an exporter protein YddG and inactivation of a permease AroP led to increased Trp pool [106,107].

The first committed enzyme of Phe and Tyr biosynthesis is CHA mutase (CM or AroQ), which converts CHA to prephenate. Prephenate is subsequently converted to Phe via phenylpyruvate or converted to Tyr via *p*-Hpp in most microorganisms [91,108]. In plant, the major route of Phe and Tyr biosynthesis occurs via an intermediate metabolite arogenate [89]. CM was only found to be feedback-inhibited by Phe, Tyr, or Trp in a few cyanobacterial strains [109]. However, CM-prephenate dehydrogenase (TyrA) is inhibited by Tyr and CM-prephenate dehydratase (PheA) is inhibited by Phe in *E. coli* [110,111,112]. Blocking completive Phe and Tyr biosynthetic pathways by deletion of *pheA* and *tyrA* could lead to the enhanced production of Trp [99]. However, this strategy may affect the biosynthesis of *p*-Hpp.

## 5. Suggested Strategies for Engineering Scytonemin Production

### 5.1. The Host for Scytonemin Production

The host selection is the first and most important consideration for metabolic engineering. Scytonemin is solely produced in some cyanobacterial species, particularly those exposed to high solar radiation. They are natural ideal hosts for metabolic engineering for scytonemin production. The exploitation of these photosynthetic microorganisms is required to overcome the obstacles of mass cultivation and genetic manipulation. The complete gene clusters or genes for scytonemin biosynthesis were first elucidated in the model cyanobacterium *N. punctiforme* PCC 73102. Other model cyanobacteria, such as *Synechocystis* PCC 6803 and *Nostoc* PCC 7120, do not synthesize scytonemin due to the lack of complete scytonemin-biosynthetic genes [36,67]. For example, the homologous genes of *Npun_R1268* and *Npun_R1263* are absent in the *Nostoc* PCC 7120 genome. The supplementation of absent genes is a potential strategy to exploit them as metabolic engineering hosts. Non-photosynthetic microorganisms such as *E. coli* are also proper selection to engineer the production of scytonemin, but only the scytonemin monomer could be obtained so far [69]. A further processing for oxidative dimerization is necessary. In cyanobacteria, this step is realized after the export of the scytonemin monomer to the periplasm with the help of Ebo proteins [64]. Therefore, the complete biosynthesis of the scytonemin dimer in *E. coli* is still a challenge.

*N. flagelliforme*, a close relative of *N. punctiforme*, was recently sequenced [113]. It is a promising scytonemin producer [8,13]. It is easy to be cultivated in liquid suspension and can produce lots of exopolysaccharides. The dense exopolysaccharide matrix provides an ideal environment for accommodating scytonemin. In addition to *COO91_00781*, *COO91_00780*, and *COO91_00775* (the homologous genes of *Npun_R1268*, *Npun_R 1269*, and *Npun_R1274*, respectively), other scytonemin-biosynthetic genes all have paralogous genes in the *N. flagelliforme* genome. Its genetic manipulation with CRISPA system was recently established in our laboratory, which will benefit its exploitation in metabolic engineering. 

### 5.2. Carbon Flux Modification 

High productivity of a target metabolite requires optimizing the redirection of carbon flux through primary and secondary metabolisms. Useful strategies for scytonemin production may include: (1) Engineering the CBB cycle or high-density cultivation to enhance photosynthetic carbon fixation and biomass increase [75,114]; (2) re-directing the metabolic flux towards the shikimate and aromatic amino acid pathway, such as improving the availability of the precursors PEP and E4P, relieving the rate-limiting enzymatic reactions, removing the transcriptional and allosteric regulations, and disruption of consumptive bypasses [72,115]; and (3) enhancing the transcription of *scy* and *ebo* gene clusters or the critical genes for the direct scytonemin biosynthesis [63,64], especially rewiring or optimizing the above-mentioned four “Modules”. Random mutagenesis is also an important technology for relieving product inhibition or ameliorating metabolic suitability in hosts [73,116]. 

### 5.3. Cultivation and Harvest Technology 

Cultivation and harvest technologies are also important for final target metabolite yield. Useful strategies may include: (1) Decoupling growth and production or so-called two-stage cultivation strategy. This strategy was usually adopted in algal metabolite production [117,118,119]. (2) Enhancing the product export, accumulation, and collection. It is particularly beneficial in industrial production. Like developing the fatty acid secreting system in cyanobacteria [120], realizing the release of scytonemin from the exopolysaccharide matrix to the surface of liquid culture is also an attractive technology. (3) Adopting stressful conditions during the whole culture process. The biosynthesis of metabolites can be triggered by a number of abiotic stresses [121]. A drawback is that abiotic stresses often slow down or restrain the biomass increase and thus an elaborate balance between the stress factor and cell growth should be established. (4) Supplementing external substrates or elicitor substances from concomitant bacteria [122]. 

## 6. Perspective

Scytonemin is a cyanobacterial secondary metabolite with a high potential market value. Metabolic engineering techniques will provide one of the key solutions for achieving cost-effective production of scytonemin in the near future. Previous engineering in the *E. coli* strain can only produce the scytonemin monomer. Along with the recent elucidation of its biosynthesis mechanism and related carbon flux-rewiring technologies, highly efficient biosynthesis of scytonemin in suitable cyanobacterial hosts, including *N. flagelliforme* and *N. punctiforme*, is greatly expected as an important starting point.
